# A liquid fraction of extracellular matrix inhibits glioma cell viability *in vitro* and *in vivo*

**DOI:** 10.18632/oncotarget.28203

**Published:** 2022-02-21

**Authors:** Mark H. Murdock, George S. Hussey, Jordan T. Chang, Ryan C. Hill, David G. Nascari, Aparna V. Rao, Kirk C. Hansen, Lesley M. Foley, T. Kevin Hitchens, Nduka M. Amankulor, Stephen F. Badylak

**Affiliations:** ^1^Department of Surgery, University of Pittsburgh, Pittsburgh, PA, USA; ^2^McGowan Institute for Regenerative Medicine, University of Pittsburgh, Pittsburgh, PA, USA; ^3^Department of Biochemistry and Molecular Genetics, University of Colorado Denver, Aurora, CO, USA; ^4^Animal Imaging Center, School of Medicine, University of Pittsburgh, Pittsburgh, PA, USA; ^5^Department of Neurobiology, University of Pittsburgh, Pittsburgh, PA, USA; ^6^Department of Neurological Surgery, University of Pittsburgh, Pittsburgh, PA, USA

**Keywords:** extracellular matrix, brain cancer, glioma treatment, dynamic reciprocity, tissue organization field theory

## Abstract

Suppressive effects of extracellular matrix (ECM) upon various cancers have been reported. Glioblastoma multiforme has poor prognosis and new therapies are desired. This work investigated the effects of a saline-soluble fraction of urinary bladder ECM (ECM-SF) upon glioma cells. Viability at 24 hours in 1, 5, or 10 mg/mL ECM-SF-spiked media was evaluated in primary glioma cells (0319, 1015, 1119), glioma cell lines (A172, T98G, U87MG, C6), and brain cell lines (HCN-2, HMC3). Viability universally decreased at 5 and 10 mg/mL with U87MG, HCN-2, and HCM3 being least sensitive. Apoptosis in 0319 and 1119 cells was confirmed via NucView 488. Bi-weekly intravenous injection of ECM-SF (120 mg/kg) for 10 weeks in Sprague-Dawley rats did not affect weight, temperature, complete blood count, or multi-organ histology (*N* = 5). Intratumoral injection of ECM-SF (10 uL of 30 mg/mL) at weeks 2–4 post C6 inoculation in Wistar rats increased median survival from 24.5 to 51 days (hazard ratio for death 0.22) and decreased average tumor volume at time of death from 349 mm^3^ to 90 mm^3^ over 10 weeks (*N* = 6). Mass spectrometry identified 2,562 protein species in ECM-SF, parent ECM, and originating tissue. These results demonstrate the suppressive effects of ECM on glioma and warrant further study.

## INTRODUCTION

The extracellular matrix (ECM) represents a major portion of the tissue microenvironment and is composed of functional and structural molecules such as cytokines, growth factors, proteoglycans, collagens, and matrix-bound nanovesicles, among others [1–3]. The microenvironment markedly influences cell behavior, survival, gene expression, and phenotype and is a major factor in the dynamic and continuous cell-matrix crosstalk referred to as “dynamic reciprocity” [4, 5].

The tissue organization field theory (TOFT) supposes that metaplastic and neoplastic transformation of cells is directly related to changes in the microenvironment/ECM [6, 7]. Therefore, if this theory is accurate, manipulation of the tumor microenvironment could modulate neoplastic cell behavior or survival. The specific components of the ECM milieu that modulate cell phenotype are only partially understood.

Several studies have documented the effects of mammalian non-neoplastic ECM upon neoplastic cells *in vitro* and *in vivo*. These studies have included several types of cancer including breast, urinary bladder, prostate, colon, skin, and esophageal, among others [8–15]. A recent study showed the beneficial therapeutic effects of an ECM hydrogel derived from normal esophageal tissues upon dysplastic esophageal mucosa (i.e. Barrett’s esophagus) in which metaplastic cells regressed to a squamous epithelial phenotype in a canine model [16]. Importantly, a study by the same group found increased apoptosis in one metaplastic and two neoplastic cell lines exposed to urinary ladder matrix ECM [13].

The objective of the present study was to determine the *in vitro* and *in vivo* effects of a saline-soluble fraction of ECM harvested from non-neoplastic porcine urinary bladder (ECM-SF) upon glioblastoma cells. ECM from porcine urinary bladder is FDA approved for many clinical applications related to wound healing and due to the source material’s ease of acquisition and simple decellularization process it is a common research material in many laboratories. The isolation of ECM from native porcine bladder involves a selective reduction in composition and elimination of cellular debris. The further processing of ECM to ECM-SF represents an enrichment of saline-soluble species, excluding saline-insoluble macromolecules. Utilizing saline-soluble fraction allowed ECM-SF to be injected into a glioma tumor without resection, thereby allowing alteration instead of removal of the tumor microenvironment. A secondary objective of the present study was to compare the proteomic composition of the native bladder, ECM, and ECM-SF as a first step toward identifying potential molecules of interest.

It was hypothesized that introducing ECM-SF into the tumor microenvironment would have the potential to modulate the glioma cell phenotype and thereby prolong survival or reduce tumor volume in an animal model of glioblastoma. We further hypothesized that ECM-SF would contain a distinct proteomic composition from that of the parent ECM.

## RESULTS

### Saline-soluble fraction of ECM decreased glioma cell viability

**Figure 1:**
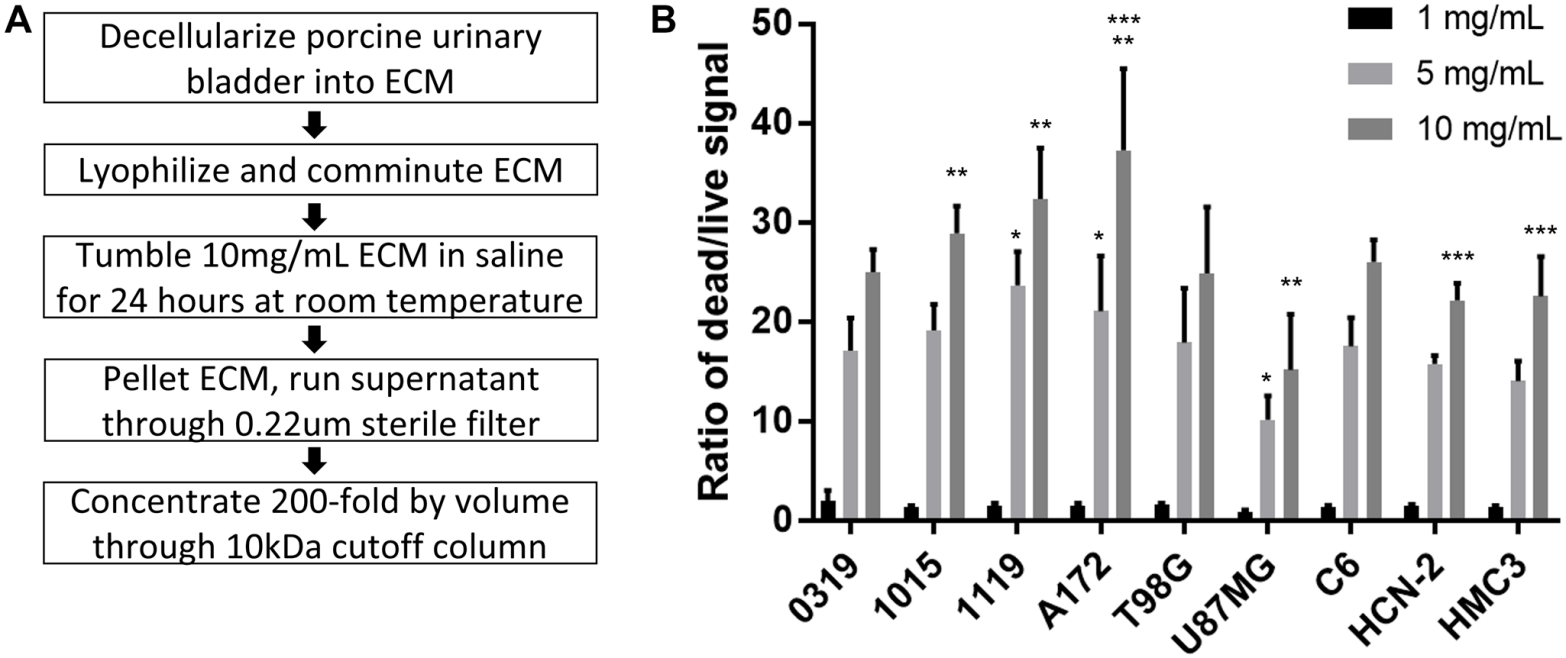
ECM-SF isolation and effect on glioma cells. Procedure for isolating and concentrating a saline-soluble fraction of ECM derived from porcine bladder (**A**). ANOVA with post hoc Tukey’s Honest Significant Difference show no difference at 1 mg/mL (*P* = 0.13), at 5 mg/mL 1119 and A172 differ from U87MG (*P* < 0.01), and at 10 mg/mL 1015, 1119, and A172 differ from U87MG, and A172 differs from HCN-2 and HMC3 (*P* < 0.01) (**B**).

Porcine urinary bladder was decellularized into ECM and then lyophilized, comminuted, tumbled in saline, centrifuged, filtered, and concentrated 200-fold by volume into ECM-SF (Figure 1A). ECM-SF was clear and colorless with viscosity comparable to water and protein content of approximately 25–30 mg/mL as measured by A280 absorbance. At 1 mg/mL cell groups averaged dead-to-live ratios between 0.9- to 2.0-fold that of the media control (Figure 1B). At 5 mg/mL cell groups averaged ratios between 10- to 23-fold, and at 10 mg/mL cell groups averaged ratios between 15- and 37-fold compared to the media control. ANOVA indicated no differences within the 1 mg/mL treatment group (*P* = 0.13) whereas at 5 mg/mL the 1119 and A172 dead-to-live ratios were significantly higher than that of U87MG (*P* < 0.01). At 10 mg/mL the 1015 and 1119 ratios were significantly higher than that of U87MG and the A172 dead-to-live ratio was significantly higher than those of non-neoplastic HCN-2 and HMC3 (*P* < 0.01).

### ECM-SF induced apoptosis in glioma cells

**Figure 2:**
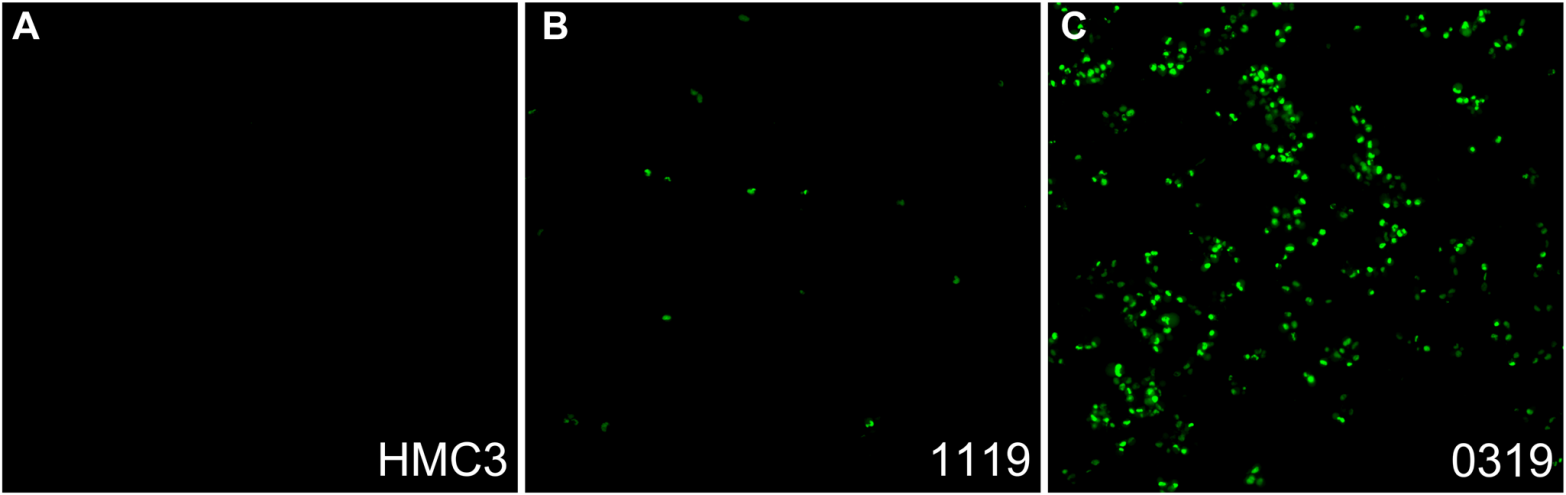
Caspase activity of glioma cells in ECM-SF spiked media. After 12 hours in media containing 3 mg/mL ECM-SF and 1.5 uM NucView there is no fluorescence from HMC3 cells (**A**), some presence of nuclear fluorescence from 1119 cells (**B**), and a high presence of nuclear fluorescence from 0319 cells (**C**). Static data shown in graphical form is at the 12-hour timepoint.

Time-lapse video showed non-neoplastic HMC3 cells emitting virtually no fluorescence over 12 hours in media spiked with 3 mg/mL ECM-SF and 1.5 uM NucView 488, a reagent fluorescent upon cleavage by active caspase-3 (Figure 2A, Supplementary Video 1). 1119 and 0319 cells, however, showed increasing amounts of nuclear fluorescence throughout the 12-hour period with 0319 cells showing an appreciably higher amount of fluorescence (Figure 2B and 2C, Supplementary Videos 2 and 3).

### Systemically injected ECM-SF did not alter hematology metrics

**Figure 3:**
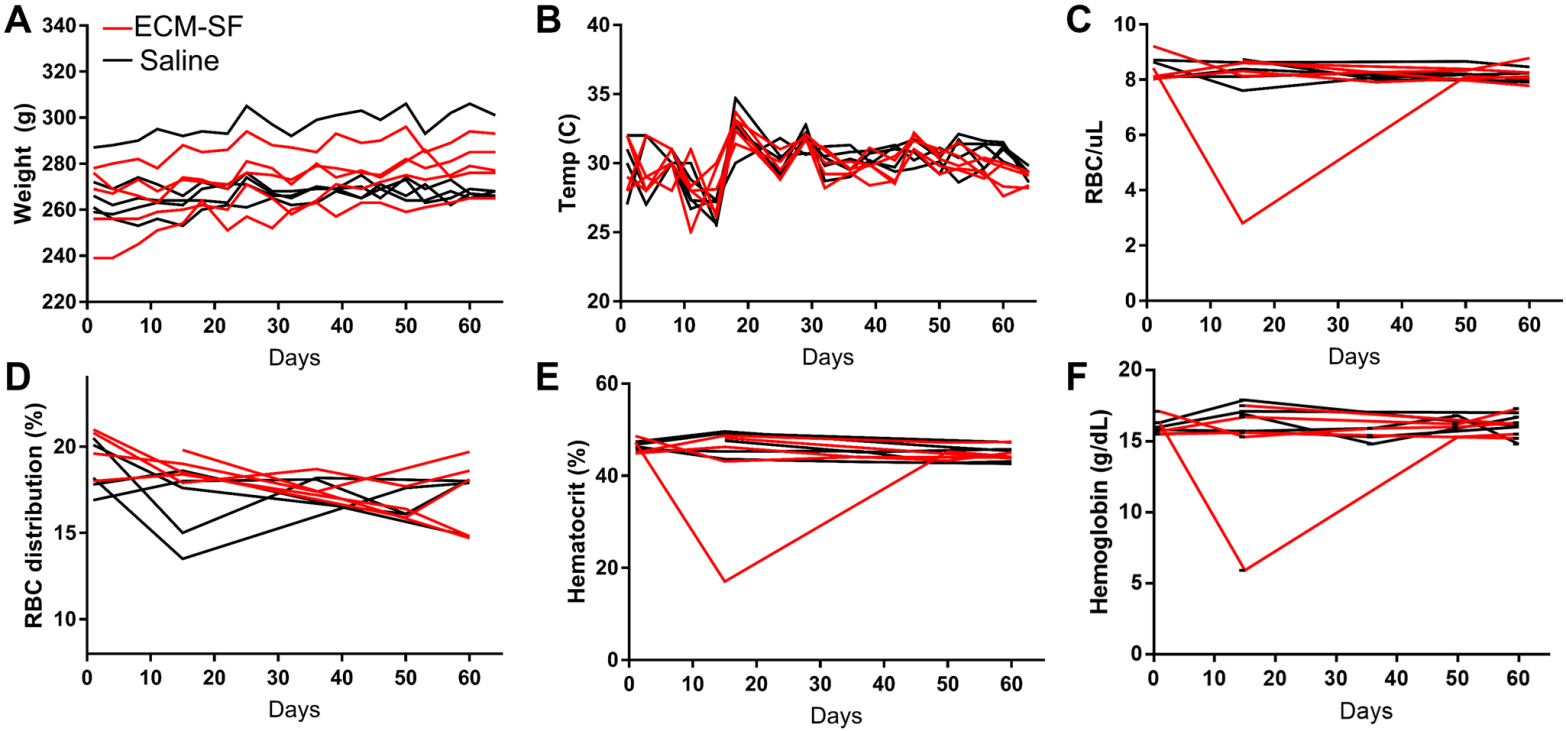
Systemic response to ECM-SF injections. No differences were found in weight (**A**) or temperature (**B**) between the ECM-SF and saline-treated groups. Hematology metrics for the two groups were normal with respect to red blood cell count (**C**), red blood cell distribution (**D**), hematocrit (**E**), and hemoglobin levels (**F**).

There were no significant differences in weight (*P* = 0.8) or temperature (*P* = 0.18) between the ECM-SF and saline-treated groups (Figure 3A, 3B). The ECM-SF group did gain more weight per animal on average (15.6 grams) than the saline group (4.6 grams), but the difference was not significant (*P* = 0.08). Hematology metrics for the two groups were normal with respect to red blood cell count (Figure 3C, *P* = 0.46), red blood cell distribution (Figure 3D, *P* = 0.20), hematocrit (Figure 3E, *P* = 0.32), and hemoglobin levels (Figure 3F, *P* = 0.30). H&E staining of lung, spleen, liver, heart, brain, and kidney appeared normal for both groups.

### Brain tissue tolerated ECM-SF which was likely retained up to 24 h

**Figure 4:**
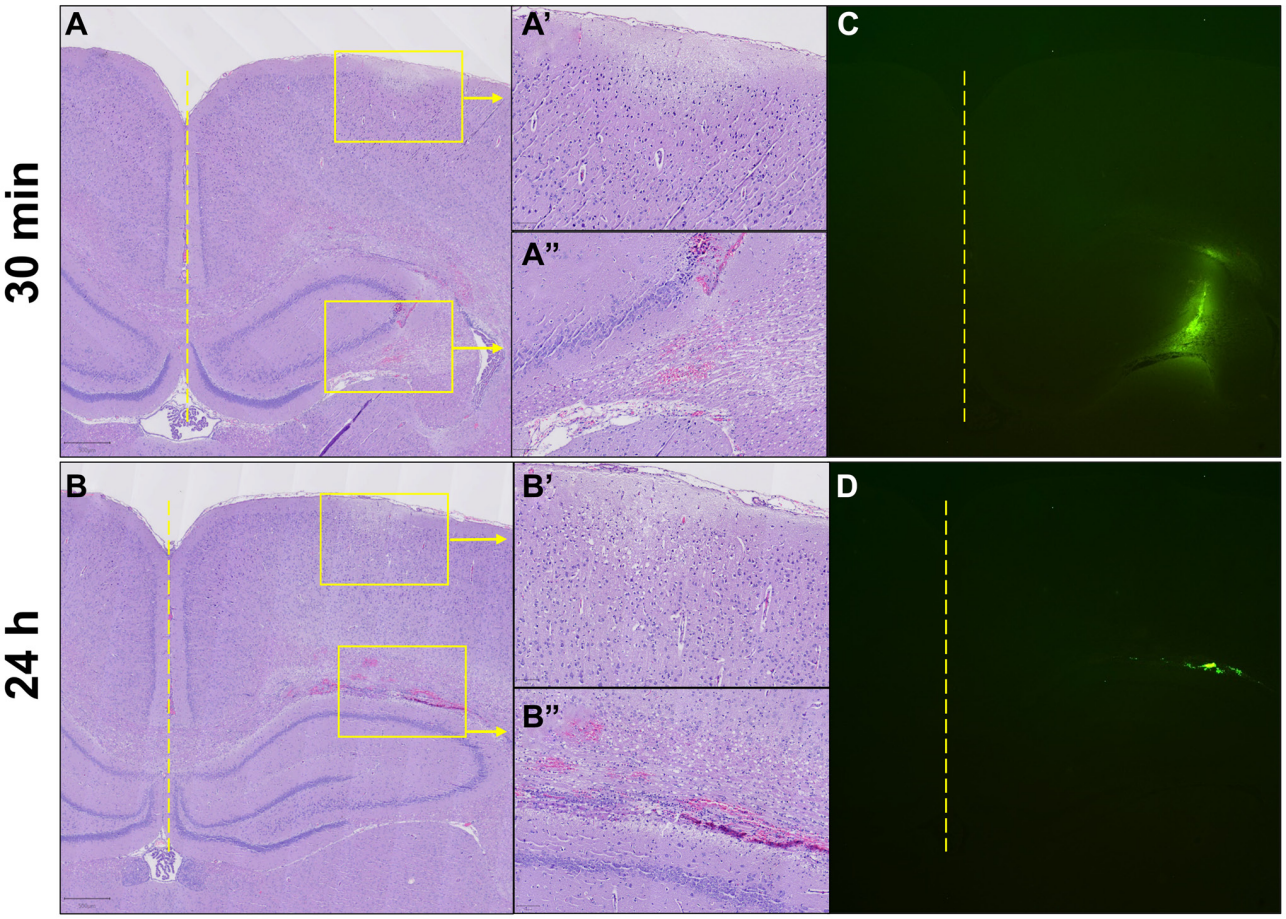
Acute intracranial cellular response to ECM-SF and retention of FITC-dextran-spiked ECM-SF after direct intracranial injection. No cellular infiltration was observed in response to the injection at 30 minutes (**A**–**A’’**, 2× and 10×) or 24 hours (**B**–**B’’**, 2× and 10×). FITC signal approximating injection diffusion and location at 30 minutes occupied ~22 times more area than at 24 hours and displayed a gradually fading periphery compared to the sharply disappearing edge of the 24-hour signal. (**C**, **D**). Yellow dotted line indicates the midline of the brain, yellow boxes indicate magnified areas.

H&E staining showed no mononuclear cellular infiltration in response to the direct intracranial injection at 30 minutes (Figure 4A–A’’) or 24 hours (Figure 4B–B’’). FITC traces approximating injection diffusion and location were clearly visible in the injection area at both timepoints (Figure 4C and 4D). The FITC signal at 30 minutes occupied approximately 22 times more area than at 24 hours (20,903 vs. 958 pixels) and displayed a gradually fading periphery compared to the sharply disappearing edge of the 24-hour signal.

### ECM-SF increased lifespan in glioblastoma model which correlated with lower tumor volume

**Figure 5:**
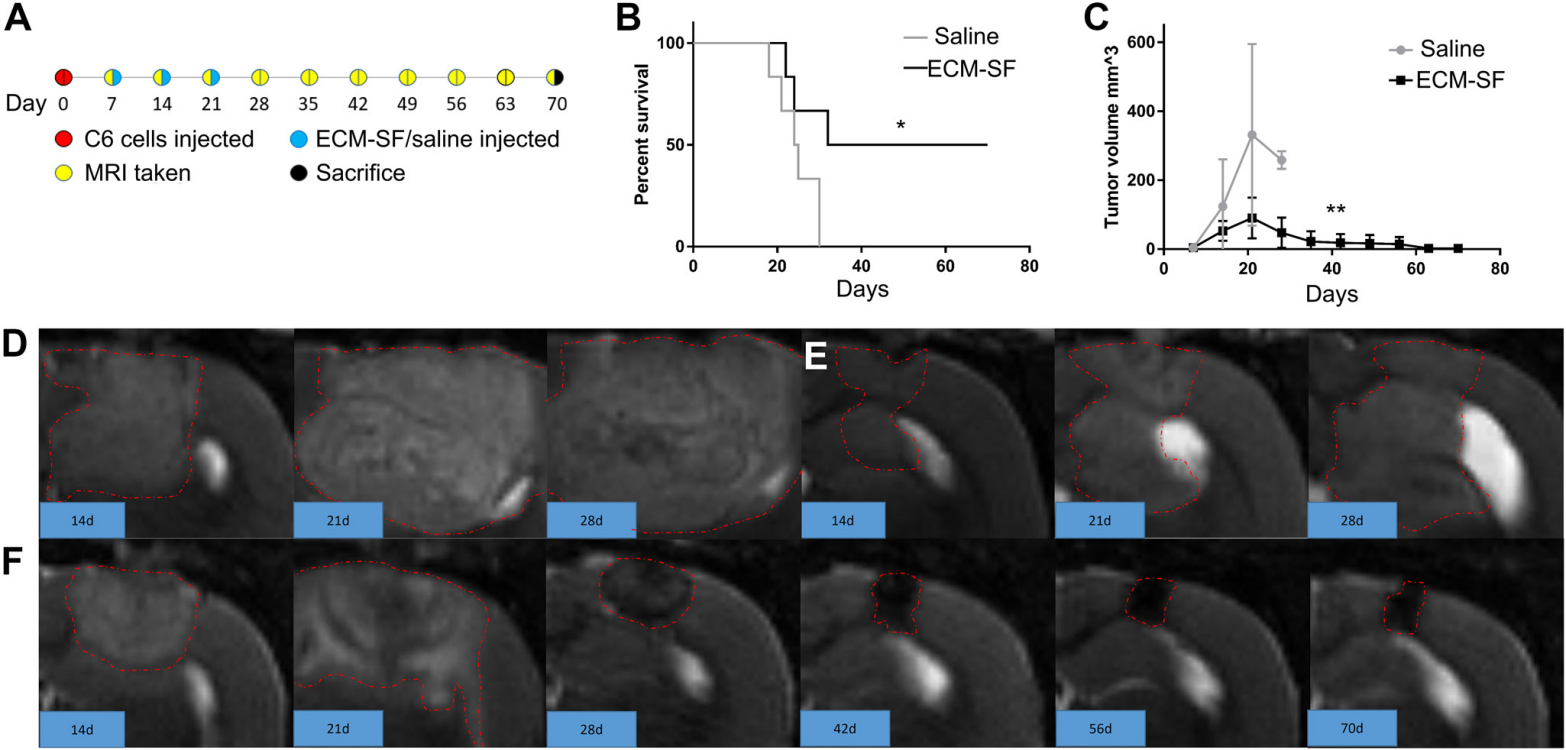
Pre-clinical model of treatment of glioblastoma with ECM-SF. (**A**) The 70-day experimental outline including inoculation of Wistar rats with C6 cells, three weekly injections of ECM-SF or saline, and weekly MRI scans until day 70. (**B**) The survival curves of ECM-SF vs. saline-treated animals (*N* = 6). Curve comparison with the Gehan-Breslow-Wilcoxon method shows no significant difference (*P* = 0.086) whereas the Mantel-Cox method shows significance (*P* = 0.046). (**C**) The average tumor volumes of ECM-SF vs. saline-treated animals over 70 days. Error bars indicate standard deviation. Comparison with unpaired *t* test shows significance (*P* = 0.0057). Representative MRI images of primary tumor at days 14, 21, and 28 of (**D**) a saline-treated rat and (**E**) an ECM-SF treated rat that died in week 4, as well as weekly images through day 70 of an ECM-SF treated rat that was electively sacrificed at day 70. (**F**) Tumor appears to have resolved into a small necrotic core with no recurrence of tumor mass. Tumor border outlined in a red dotted line.

C6 cells were injected at day 0 and ECM-SF or saline injections were given intratumorally at days 7, 14, and 21 (Figure 5A). Of the six saline-treated animals, three died at days 18, 21, and 30, and three were sacrificed at days 24, 25, and 30 in accordance with IACUC tumor burden guidelines. Three ECM-SF treated animals were taken off protocol at days 22, 24, and 32. The three remaining ECM-SF treated animals survived until day 70 and presented as bright, alert, and responsive before electively being taken off protocol. ECM-SF increased median survival from 24.5 to 51 days and the hazard ratio for death was 0.22 (95% CI, 0.05 to 0.97). Survival curves were compared using both the Gehan-Breslow-Wilcoxon, which places more weight on early time points, and the Mantel-Cox method, which gives equal weight to all timepoints, resulting in *P* = 0.086 and *P* = 0.046, respectively (Figure 5B). The saline-treated rats had an average tumor volume of 349 mm^3^ at time of death compared to 90 mm^3^ for the ECM-SF treated animals. Unpaired *t*-test analysis showed significant differences between the tumor volumes of the two groups (Figure 5C, *P* = 0.0057). The three ECM-SF treated rats that survived to day 70 had decreasing tumor volumes over time (Figure 5C). Representative MRI images show tumor characteristics over time (Figure 5D–5F). The six saline-treated animals that died by day 30 had large solid tumors which grew rapidly (Figure 5D). The three ECM-SF treated animals which died by day 32 had smaller, slower growing tumors (Figure 5E). The three ECM-SF treated animals which survived to day 70 had smaller, slower growing tumors which resolved into a necrotic core (approximately 1.7 mm^3^) by day 70 (Figure 5F).

### ECM-SF has a distinct composition compared to parent ECM/tissue

**Figure 6:**
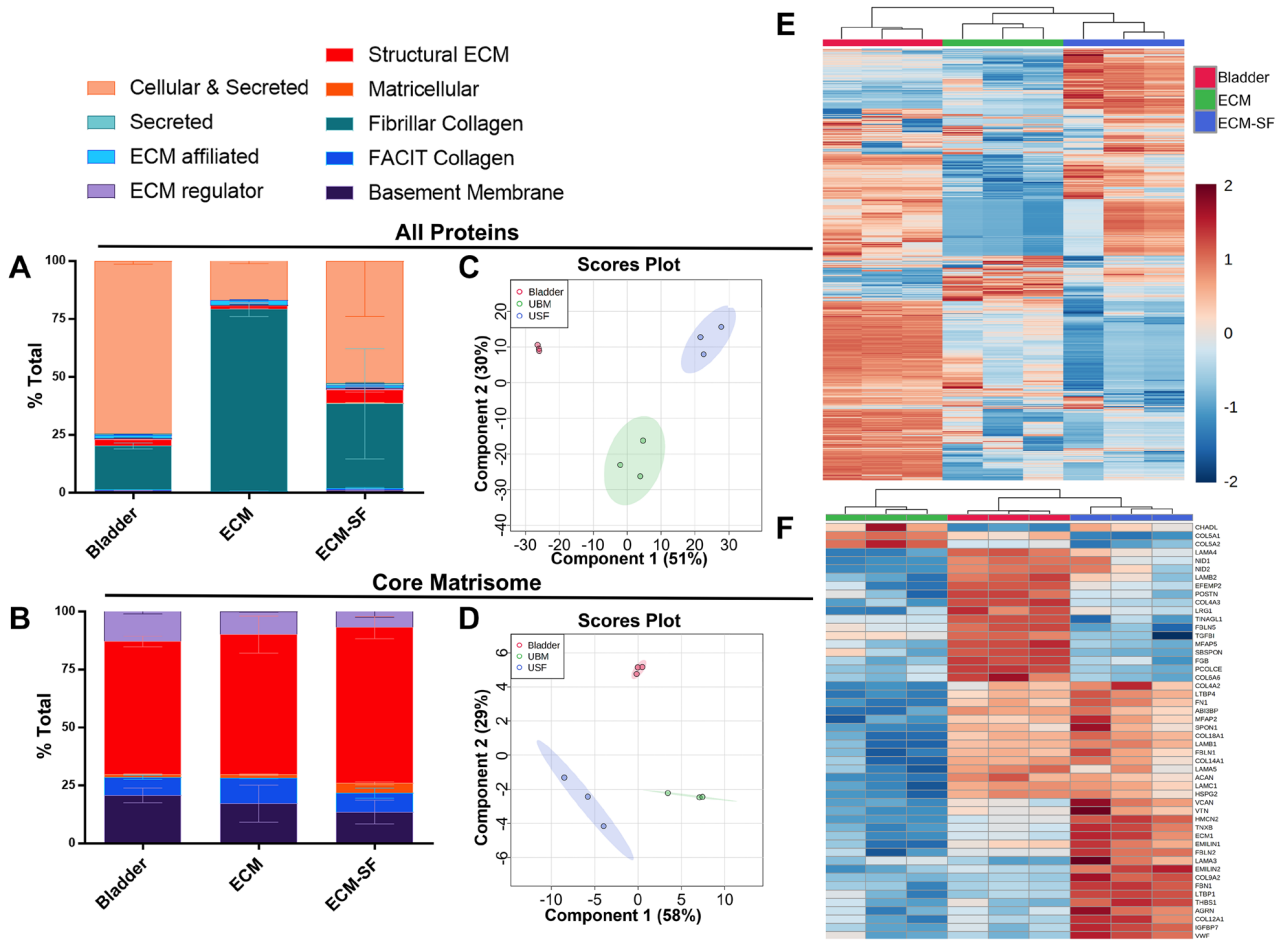
Global and targeted proteomic analysis of bladder, ECM, and ECM-SF. Sorting by functional categories reveals the majority to be Cellular & Secreted, or Fibrillar Collagen (**A**). Each source material contains a similar core matrisome composition (**B**). Principal component analysis shows all proteins and core matrisome proteins from source materials to be distinct compositional groups (**C**, **D**). 2,562 distinct protein species were identified: 2,373 in bladder, 2,010 in ECM, and 1,017 in ECM-SF (**E**). The top fifty differentially expressed core matrisome proteins from each source material were identified (**F**).

Native porcine bladder, decellularized ECM, and saline-soluble ECM-SF were subjected to global and targeted mass spectrometry analysis. A total of 2,562 distinct protein species (*N* = 3 biological and technical replicates) were identified. Of these proteins, bladder contained 2,373 protein species, ECM contained 2,010 species, and ECM-SF contained 1,017 species. Proteins were sorted into functional categories (Figure 6A) or core matrisome proteins only (Figure 6B). All proteins and core matrisome proteins from bladder, ECM, and ECM-SF were shown to be members of distinct compositional groups via principal component analysis (Figure 6C, 6D). Bladder biological replicates were shown to have the least amount of variability. Hierarchical clustering of all proteins showed biological replicates within each source material to be most related to each other, and ECM and ECM-SF were more closely related to each other than to the native bladder (Figure 6E, 6F). There were, however, many instances of differential presence of certain proteins between the biological replicates in each source material. ANOVA analysis identified the top fifty core ECM proteins showing a difference between the source materials (Figure 6F).

### ECM-SF enriches 618 proteins from parent ECM

**Figure 7:**
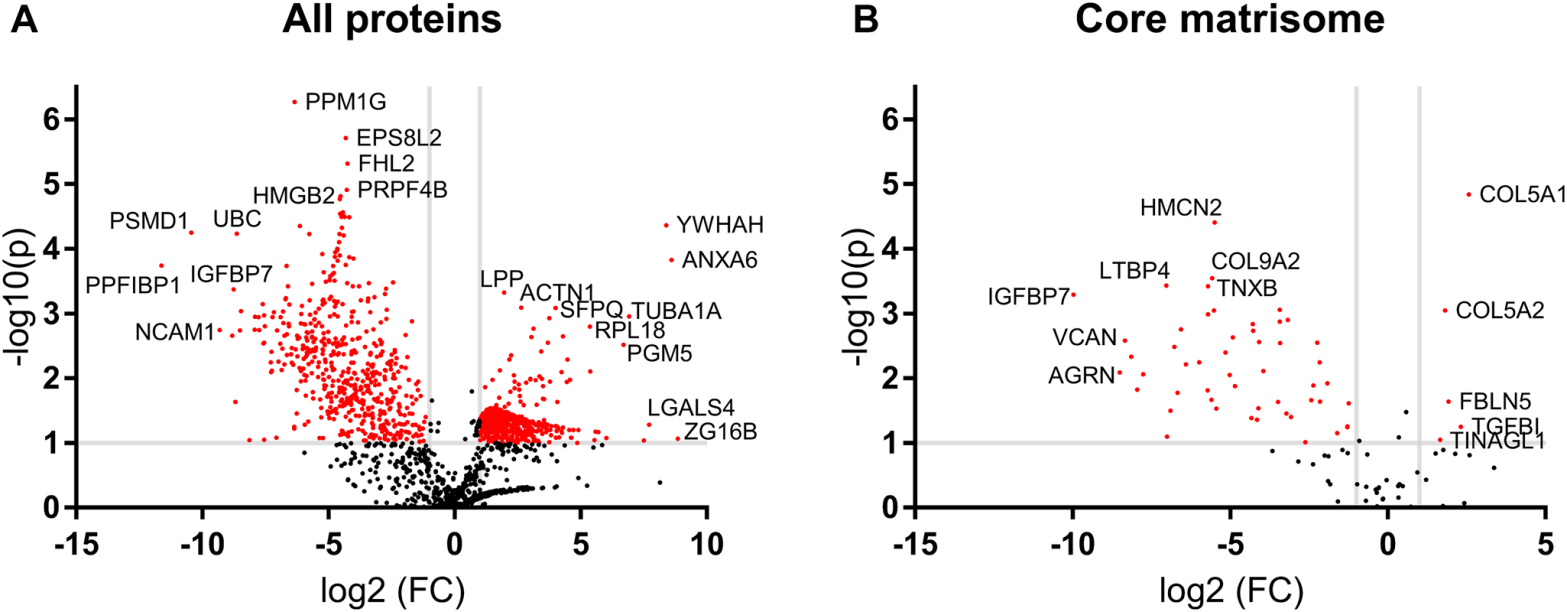
Volcano plot of ECM-SF compared to ECM. (**A**) 618 protein species were enriched two-fold or more in the ECM-SF while 528 protein species remained two-fold or higher in the parent ECM. (**B**) For the core matrisome, five protein species were enriched in the ECM-SF while 51 protein species remained two-fold or higher in the parent ECM.

A volcano plot comparing the ECM-SF to ECM proteome showed 618 proteins enriched in the ECM-SF above a 2-fold change and below a *P* value of 0.1. 528 proteins present in the urinary bladder extracellular matrix (UB-ECM) were identified with the same inclusion criteria (Figure 7A). In the core matrisome, ECM-SF contained five proteins above a 2-fold change and below a *P* value of 0.1 compared to ECM, while ECM contained 51 proteins (Figure 7B, Supplementary Table 1).

## DISCUSSION

The results of the present study show that a saline-soluble fraction of ECM can decrease glioma cell viability *in vitro* via caspase-3 mediated apoptosis, and intratumoral injection intervention can decrease glioma tumor volume *in vivo*, correlating with longer survival. ECM-SF did not elicit an acute (24 hours) cellular response in rat brain tissue, nor have deleterious effects on weight gain, temperature, or hematology metrics when administered intravenously (10 weeks). The protein profile of ECM-SF was distinct from both its parent ECM and the source tissue. Several protein species (COL5A1, COL5A2, FBLN5, TGFBI, TINAGL1) were enriched in the saline-solubilization process. The results support the findings of other ECM-based studies and the TOFT; that is, that non-neoplastic mammalian ECM contains bioactive cues that can antagonize cancer cells and their *in vivo* progression [10–14, 16]. The findings were present *in vitro* in both primary glioma cells and glioma cell lines, though with differing sensitivities. These findings led to anti-tumor results in the widely used allogeneic rat glioma model C6-Wistar.

The concept that ECM and resident cells influence each other through an ongoing, bidirectional crosstalk is called “dynamic reciprocity” [4, 5]. This dynamic process is seen in the tumor microenvironment wherein cancer cells slowly degrade, secrete, and remodel the local ECM which, in turn, can influence the gene expression of non-neoplastic cells to behave more cancer-like [8]. The opposite phenomenon also holds true; that is, that cancer cells introduced into non-neoplastic microenvironments can transform to become less cancer-like. Bissel et al. showed that breast cancer cells respond to their microenvironment and can revert from their malignant phenotype [8]. Hodde et al. found that porcine small intestinal submucosa ECM (SIS-ECM) did not hasten the formation of primary tumors and decreased the volume of recurring tumors after surgical resection [9]. Hurst et al. found that high grade metastatic bladder cancer cells grown on SIS-ECM hydrogel decreased invasion and showed more organized growth than when grown on Matrigel or collagen [10]. Saldin et al. showed that UB-ECM and esophagus-derived ECM both decreased proliferation of esophageal cancer cells and that UB-ECM differentially increased apoptosis [13]. Wolf et al. showed that a solid UB-ECM scaffold inhibited melanoma tumor formation in a macrophage-dependent manner in mice [14]. These findings are consistent with the concept of tissue organization field theory which challenges the dogma of somatic mutation theory regarding the origin of cancer [6, 7]. Somatic mutation theory is cell-centric, attributing the rise of cancer solely to the accumulation of DNA damage and mutation in cells. In contrast, TOFT is tissue-centric, ascribing the onset of cancer to aberrations in the microenvironment, of which the ECM is a major part. These concepts are not mutually exclusive. If a microenvironment can induce oncogenesis, perhaps an alternative microenvironment can modulate oncogenic behavior, or simply eradicate the affected cells. This concept begets a potential alternative approach to conventional cancer therapeutics. While the literature largely describes the transformative effect of ECM on cancer cells, the main result seen in this work is glioma cell death. This result is in line with the observations of Saldin et al. where UB-ECM increased apoptosis in esophageal cancer cells and might be due to the increased concentration of ECM components found in ECM-SF after the 200-fold reduction in volume.

Porcine urinary bladder was the source tissue for the ECM used in the present study for several reasons. It is one of the most readily available and commonly used ECM biomaterials. The decellularization process to manufacture UB-ECM is well established, relatively simple, and involves primarily mechanical removal of the tunica serosa, tunica muscularis externa, tunica submucosa, and most of the tunica muscularis mucosa to decrease cellularity [17]. Harsh decellularization processes that rely upon chemical detergents tend to deplete growth factors, and these detergents often remain in the resulting bioscaffold, skewing experimental results. ECM biomaterials derived from porcine urinary bladder are commercially available (MatriStem, ACell, Columbia, Maryland), retain bioactivity after electron beam terminal sterilization, and have been used in a clinical trial involving esophageal adenocarcinoma patients wherein the implanted bioscaffold degraded within two weeks, promoting functional remodeling of the esophagi which have remained cancer-free to date (clinicaltrials.gov Identifier: NCT02396745).

ECM is a complex structure comprised of hundreds of molecules of various size. Fractionation is a reductionist approach toward identifying molecules of interest. ECM is most commonly used therapeutically as a solid sheet, a comminuted powder, or as a hydrogel. Slivka et al. showed that UB-ECM hydrogel can be centrifuged into “structural” and “soluble” fractions, which have differential effects in regulating macrophage behavior [18]. Several advantages derive from working with a “liquid-form ECM”. A liquid form allows for the ECM components to be concentrated tens or hundreds of times, which is not possible with sheets, powders, or digested hydrogels. The liquid form allows for intratumoral injection without first resecting any tissue mass which may have future application in treatment of surgically inoperable tumors. Extraction of UB-ECM using normal saline is logical due to its physiological relevance (interstitial fluid also having 0.9% NaCl). It is known that implanted solid forms of ECM and hydrogel formulations can recruit stem/progenitor cells and modulate macrophage responses within 24 hours. Therefore, this length of time for the saline-extraction may capture many of the molecules responsible for these functional observations [19, 20]. It is believed that host degradation of implanted ECM is essential for the release of bioactive components which account for positive remodeling effects [21, 22].

It is not surprising that ECM-SF did not elicit an acute cellular response in the rat brain. UB-ECM hydrogels have been used intracranially for stroke studies without ill effect and indeed have exhibited positive tissue remodeling within the central nervous system [19, 23]. Studies have also shown UB-ECM hydrogels to recruit doublecortin-positive neural progenitor cells, support neuron viability, and stimulate axon growth [24–27]. SIS-ECM (Durasis, Cook Biotech, West Lafayette, Indiana) has been used as a dural substitute where it is claimed to remodel into native tissue. Other ECM-based materials made from pericardium or dermis are also commercially available [28]. Intravenous injection was conducted to determine the safety of this ECM formulation and allowed for future potential investigation of a systemic approach for cancer, whether prophylactic or therapeutic. Future studies will address the mechanism by which the body processes and metabolizes ECM components, the salient species that are permeable through the blood-brain-barrier, the effect upon the immune system, and toxicological studies of specific molecules.

Although the molecular mechanism(s) by which ECM-SF mitigates cancer cell growth is beyond the scope of the present study, the proteomic analysis conducted herein does shed light on possible protein effector molecules. Of the 2,562 protein species identified in the native bladder, ECM, and ECM-SF, 1,017 were contained in the ECM-SF. The volcano plot showed ECM-SF contained only five proteins enriched more than two-fold from the parent ECM which combined have a mixed effect on glioma. The expression levels of COL5A1 and COL5A2 are significantly correlated with glioma progression stage [29]. FBLN5 is downregulated in stage III and IV glioma and has been shown to inhibit glioma cell proliferation and invasion [30]. High TGFBI expression promotes proliferation and migration of glioma cells [31]. TINAGL1 is largely unstudied in the context of glioma but suppresses triple-negative breast cancer progression and metastasis [32]. Although these proteins were highlighted by the volcano plot, their role in driving the observed effects is unknown.

It is possible, and seems likely, that in addition to the direct anti-glioma effects of ECM-SF observed *in vitro* there are indirect anti-glioma effects attained through modulation of the immune system. It seems unlikely that the ECM-SF could exert direct anti-glioma effects in the C6-Wistar model throughout ten weeks when ECM-SF injections only occurred on days 7, 14, and 21. It is known that non-neoplastic ECM modulates macrophages away from an M1 activation state toward an M2-like state. Tumor associated macrophages, known as TAMs and perhaps better thought of as M(tumor activated), create an immunosuppressive microenvironment and secrete cytokines that activate anti-apoptotic programs in cancer cells [33]. Microenvironmental conditioning provided by ECM-SF may modulate TAMs away from their tumor-supportive role toward an M(ECM activated) state and allowing anti-tumor efficacy. This mechanism would explain the observation of gradually diminishing tumor mass.

There are limitations beyond those already mentioned with the present study. While the glioma cells included both primary cells and cell lines, the non-glioma cells used (HCN2 and HMC3) represent transformed immortalized cells that may not be representative of the cell population in patients. Culturing cells on 2D tissue culture plastic with growth media containing BSA has limitations and introduces variables, but this approach represented proof of concept that evolved into and justified an *in vivo* pilot study. While NucView 488 is marketed as a caspase-3 specific substrate, it is based on a DEVD sequence which means it can also be cleaved by caspase-7. However, both caspase-3 and 7 are classified as executioner caspases, direct precursors to apoptosis [34]. The non-neoplastic cell lines showed significant cell death at the 10 mg/mL of ECM-SF indicating higher concentrations need not be tested *in vitro*. While ECM materials have traditionally been reported to have excellent biocompatibility, it seems ECM-SF contains one or more highly concentrated components that can damage non-neoplastic cells if administered in sufficient amounts. These components may or may not be the same that elicit the anti-cancer effect so attempting to remove them may or may not be a useful strategy in future studies. If solid or hydrogel ECM could be concentrated to the same degree as ECM-SF, similar cytotoxic results would likely be observed as virtually all test articles become cytotoxic at sufficiently high concentrations. The FITC signal attached to 70kda dextran did not specifically track the diffusion and retention of any particular component within the ECM-SF and was meant to provide a limited approximation of the level of diffusion at early time points and where to search for a cellular response to the ECM-SF. The core matrisome of ECM-SF contained components as small as Von Willebrand factor (500 – 20,000 kDa) and as large as versican (protein core of 360 kDa), supporting speculation that some of the ECM-SF components were also retained up to 24 hours. This is supported by previous work showing that ECM components from hydrogels injected into stroke cavities in rat brains were retained at 24 hours and up to at least 12 weeks [24, 25, 35]. The spatial diffusion observed in the FITC signal after 24 hours provided the rationale for using 30 mg/mL ECM-SF in the animal model as we expected the ECM-SF to diffuse over time and wanted to maintain a sufficiently high concentration throughout the tumors to see an anti-glioma effect. Only one timepoint was included in the *in vitro* experiment but this was followed with an *in vivo* experiment which tracked tumor volume for ten weeks. In a pre-study sample size calculation with continuous endpoints, two independent samples determined a minimum of *N* = 6 for the control and treatment groups where α and β were set to 0.05 and 0.8 respectively and control and treatment group means were 150 mm^3^ and 75 mm^3^ with a standard deviation of 45 mm^3^. Control volume mean was informed by the day 28 timepoint of a similar C6 glioma model, treatment mean was conservatively informed by *in vitro* studies, and standard deviation was set conservatively to allow for biologic variability [36]. Due to the unexpected result of half of the treatment animals succumbing to their tumor burden, biological variability was higher than anticipated, resulting in a significant difference between the survival curves being found by the Mantel-Cox method (*P* = 0.045) but not the Gehan-Breslow-Wilcoxon method (0.086). ECM is derived from a natural source and requires manual processing which results in sample variability. Multiple biological replicates were included in all stages of experimentation to gather representative data. Survival curves include animals that died directly from the glioma burden as well as animals that had to be sacrificed in accordance with IACUC tumor burden guidelines. As with all proteomic analyses the list of identified protein species is not comprehensive as the dark proteome could not be represented in the results.

Intervention with liquid fractions of non-neoplastic mammalian extracellular matrix (ECM) may lead to smaller tumor volume and increased lifespan in patients with glioblastoma multiforme. Further research into the anti-cancer effects of ECM biomaterials is warranted.

## MATERIALS AND METHODS

### ECM-SF preparation

Adult porcine bladders were acquired (Tissue Source, LLC, Lafayette, IN) and urinary bladder extracellular matrix (ECM) was isolated as previously described [17]. ECM sheets were then lyophilized for 72 hours, comminuted through a 40-mesh sieve in a Thomas Scientific Wiley Mini Mill, and tumbled in a Fisher Scientific tube revolver in normal saline at 10 mg/mL for 24 hours at room temperature. ECM particles were pelleted via centrifugation at 100,000 rpm for 30 minutes and supernatant was passed through 100 um cell strainers and then 0.22-micron sterile filters. Resulting liquid was concentrated 200-fold by volume in 10kDa cutoff columns (MilliporeSigma UFC901008, Darmstadt, Germany) and protein concentration was approximated using a Nanodrop A280 measurement to verify cutoff column filter integrity. Samples were stored at −20°C until use.

### Cell origin, culture, and viability assay

Patient derived glioma cells (designated as 0319, 1015, 1119) were used before passage 20. Glioma cell lines (A172, T98G, U87MG, and C6) and non-neoplastic CNS cells (HCN2 and HMC3) were obtained from ATCC (Manassas, Virginia). ATCC routinely utilizes short tandem repeat profiling to authenticate their cell line products. All cells were cultured in high-glucose DMEM with 10% FBS and 1% pen/strep with incubation at 37°C in a 5% CO2 environment. With exception to HCN2 cells which originated from a female, all cell lines used originated from male sources. Growth media was spiked with ECM-SF at concentrations of 1, 5, and 10 mg/mL for viability experiments which were conducted in triplicate. Cells were plated at 12,000/well in 96 well plates and allowed to attach overnight before spiked media was introduced. 24 hours later cell viability was measured with LIVE/DEAD™ Viability/Cytotoxicity Kit from Invitrogen (L3224 Waltham, Massachusetts), as per manufacturer’s instructions, read with a Molecular Devices SpectraMax M2 and reported as ratio of dead signal to live signal normalized to media control.

### Time lapse videography with NucView 488 reagent

125,000 cells of 0319, 1119, and HMC3 were plated into 12-well plates and allowed to attach overnight. Wells were treated either with media spiked with 3 mg/mL ECM-SF and 1.5 uM NucView 488 Caspase-3 Reagent (Biotium), or media plus NucView 488 alone. Images were taken every 10 minutes for 12 hours and compiled in AxioVs40 (Carl Zeiss) to create time-lapse videos. Static data shown in graphical form is at the 12-hour timepoint.

### Animal experiments

Animal experiments were performed in accordance with the regulations and approval of the Institutional Animal Care and Use Committee (IACUC) of the University of Pittsburgh. Animals used for the systemic intravenous injection study were opportunistically female. Male animals were used in the GBM model as malignant brain tumors generally occur more commonly in males [37].

### Systemic intravenous injection

Adult female Sprague-Dawley rats from Envigo (Indianapolis, Indiana) 250–300 g in weight were used for the systemic intravenous injection study. Animals were randomly assigned into treatment or control groups (*N* = 5) and given either 120 mg/kg of ECM-SF or the equivalent volume in normal saline every other week for a total of five injections through the tail vein. One week prior to this and every other week afterward for a total of six times, 500 uL of blood was drawn via saphenous vein puncture into EDTA coated Sarstedt Microvette tubes (Sarstedt Inc 20.1341.102, Numbrecht, Germany). Complete blood count was performed by Marshfield Labs (Marshfield, WI) and red blood cell count, red blood cell distribution, hemoglobin, and hematocrit are reported. Weight and temperature for each animal was monitored and recorded weekly. Rat temperature was monitored using a small rodent infrared thermometer from Braintree Scientific, Inc (IR-B153, Braintree, Massachusetts).

### FITC-dextran spiked ECM-SF intracranial injection

ECM-SF was spiked with 1 mg/mL 70 kda FITC-dextran (Sigma Aldrich 46945, Cleveland, Ohio). Adult male Wistar rats 250–300 g in weight (*N* = 4, Charles River Laboratories) were anesthetized with ketamine/xylazine (90/15 mg/kg intraperitoneal injection) and placed in a stereotaxic frame with 1–3% isoflurane and oxygen flowing from a nose cone. Skin was shaved and a 5 mm incision was made, allowing a burr hole to be drilled 2 mm lateral to the midline on the right side and 1 mm posterior to the bregma. A 500 uL Hamilton syringe tipped with a 25-gauge needle and attached to a syringe pump (Harvard Apparatus PHD 2000, Holliston, Massachusetts) delivered FITC-dextran spiked ECM-SF at a rate of 1uL/minute for 10 minutes at a depth of 2 mm below the brain surface. After a 5-minute equilibration period the syringe was slowly removed, the skin was sutured and glued, and the animal was recovered. Two animals were sacrificed at 30 minutes post injection, and two were sacrificed at 24 hours post injection. Sacrifice was achieved by CO2 asphyxiation followed by cervical dislocation, and brains were immediately harvested and placed in 10% neutral buffered formalin. Slides were imaged with a Motic EasyScan Pro 6 slide scanner and images exported using QuPath 0.3.2.

### Intracranial glioma model

Adult male Wistar rats 250–300 g in weight were used for the intracranial glioma model. Animals were randomly assigned into control or treatment groups (*N* = 6 each). C6 glioma cell suspensions at a density of 1 × 10^6^ cells per 10 uL in PBS were prepared fresh from mid-confluent cultures to maximize viability upon inoculation. C6 inoculum was administered via a stereotaxic frame and syringe pump as described above. At 7 days post operation and every week thereafter until day 70, animals were subjected to MRI. At days 7, 14, and 21 post inoculation animals received 10 uL of either normal saline or 30 mg/mL ECM-SF at the same stereotaxic coordinates and flow rate as the tumor inoculation. At 70 days animals were sacrificed by CO2 asphyxiation and cervical dislocation.

### MRI analysis

Rats were anesthetized via a nose cone with 1.5–2.5% isoflurane and O2. The rats were positioned on an animal bed with a stereotaxic head holder and placed in the scanner. A rectal temperature probe was used for monitoring and maintenance of temperature at 37.0 ± 0.5°C using a warm air heating system. Respiration was monitored (SA Instruments, Stonybrook, New York). MRI was performed using a 7T/30-cm AVIII spectrometer (Bruker Biospin, Billerica, Massachusetts) equipped with a 12 cm gradient set and using an 86 mm quadrature RF transmit volume coil, a 2-channel receive surface RF coil and Paravision 6.0.1. A T2-weighted RARE sequence was used to visualize the glioma in both axial and coronal orientations, with the following parameters (axial): repetition time (TR)/echo time (TE) = 5000/80 ms, field of view (FOV) = 35 × 35 mm, acquisition matrix = 192 × 192, 25 slices with a slice thickness of 1 mm, 2 averages, and a RARE factor = 8. The coronal images used the same parameters except 13 slices were used. Tumor volumes were calculated by manual segmentation in a blinded fashion using DSI Studio software (http://dsi-studio.labsolver.org/).

### Mass spectrometry

Three biological replicates of lyophilized, comminuted native bladder, ECM, and liquid ECM-SF were prepared as described above. Protein was extracted from milled samples as previously described [38]. Briefly, bladder and ECM samples were homogenized with a bead beater in three rounds of a CHAPS/high salt buffer followed by successive extractions with guanidine-HCl, and hydroxylamine-HCl. Approximately 30 ug of protein from each fraction and 30 ug of protein from ECM-SF was then trypsin digested using a filter-aided sample preparation (FASP) approach and C18 cleanup. Liquid chromatography tandem mass spectrometry (LC-MS/MS) analysis was conducted as previously described [38]. Briefly, samples were analyzed in triplicate by a nano-UHPLC-MS/MS on an Easy-nLC1200 coupled with an orbitrap Fusion Lumos Tribrid (Thermo Fisher Scientific, Waltham, Massachusetts) mass spectrometer operating in positive ion mode. Approximately 3 ug of total protein was directly loaded onto an in-house packed 100 um i.d. × 250 mm fused silica column packed with CORTECS C18 resin (2.7 um). Samples were run at 250 nL/min over a 90 min linear gradient from 4–28% acetonitrile in 0.1% formic acid. MS data acquisition was identical to those previously described [38]. Protein identification and label free quantification was done with Proteome Discoverer Software package (v2.4). Database searching was done using Mascot (v2.5) with peptide spectral matches mapped against the Uniprot *Sus Scrofa* database. Mass tolerances were set to +/− 10 ppm for parent ions, and +/− 25 ppm for fragment ions. Mixed C-terminal N (hydroxylamine specificity) and trypsin were used as cleavage rules, allowing for 2 missed cleavages. Asn and Gln deamidation, Met oxidation, Pro hydroxylation, Gln to Pyro-Glu were set as variable modification with Cys carbamidomethylation set as a fixed modification. False discovery rates were calculated using Percolator with peptide and protein identifications requiring an FDR of less than 1%.

### Statistical analysis

Data statistical analysis was performed using GraphPad Prism 7.03 software. Mantel-Haenszel approach for hazard ratio and confidence interval of Kaplan-Meier survival curve was reported. Both Gehan-Breslow-Wilcoxon and Mantel-Cox methods were used for comparison of survival curves. Student’s *t*-test was used to compare groups of two. One-way ANOVA and Tukey’s honest significant difference were used to find differences in groups of more than two. *P*-values lower than 0.05 were considered significant. Appropriate numerical data are shown as mean ± standard deviation. Heat maps plotted protein species relative abundance along -2- to 2-fold change. Volcano plots used cutoff criteria of *P* = 0.1 and increased presence by at least a 2-fold ratio.

## SUPPLEMENTARY MATERIALS










