# Supplementary material for: A liquid fraction of extracellular matrix inhibits glioma cell viability in vitro and in vivo
Source: Oncotarget. 2022 Feb 21;13:426–38. doi: 10.18632/oncotarget.28203 (PMC8860176; doi:10.18632/oncotarget.28203)
Supplement: Supplementary file 1 [file oncotarget-13-28203-s001.pdf]

A liquid fraction of extracellular matrix inhibits glioma cell viability *in vitro* and *in vivo*

SUPPLEMENTARY MATERIALS

Supplementary Table 1: Top fifty differential core matrisome protein species for reference. See Supplementary Table 1

Supplementary Video 1: A time-lapse microscopic recording, showing non-neoplastic HMC3 cells emitting virtually no fluorescence over 12 hours in media spiked with 3 mg/mL ECM-SF and 1.5 uM NucView 488 reagent. See Supplementary Video 1

Supplementary Video 2: A time-lapse microscopic recording, showing 1119 primary human glioma cells emitting a significant amount of nuclear fluorescence over 12 hours in media spiked with 3 mg/mL ECM-SF and 1.5 uM NucView 488 reagent. See Supplementary Video 2

Supplementary Video 3: A time-lapse microscopic recording, showing 0319 primary human glioma cells emitting a high amount of nuclear fluorescence over 12 hours in media spiked with 3 mg/mL ECM-SF and 1.5 uM NucView 488 reagent. See Supplementary Video 3
